# Nudging against consent is effective but lowers welfare

**DOI:** 10.1038/s41598-024-65122-0

**Published:** 2024-06-27

**Authors:** Mollie Gerver, Sanchayan Banerjee, Peter John

**Affiliations:** 1 Department of Political Economy, King’s College London, London, UK; 2 Institute for Environmental Studies, Vrije Universiteit Amsterdam, Amsterdam, The Netherlands

**Keywords:** Nudge, Autonomy, Effectiveness, Consent, Ethics, Psychology, Human behaviour

## Abstract

Behavioural nudges are often criticised because they “work best in the dark”. However, recent experimental evidence suggests that the effectiveness of nudges is not reduced when they are delivered transparently. Most people also endorse transparent nudges. Yet, transparent nudging may undermine human autonomy—a minority may oppose to being nudged and feel manipulated, even if they know what is happening. We propose an alternative way of maintaining autonomy that is not reducible to transparency: individuals can be asked if they consent in advance to being nudged. To assess whether consensual nudges are effective, we ask consent from 1518 UK citizens to be nudged. Subsequently, we default all participants into donating to a charity of their choice, irrespective of self-reported consent. We find that the default nudge is equally effective for both consenting and non-consenting individuals, with negligible difference in average donations. However, non-consenting individuals report higher levels of resentment and regret and lower levels of happiness and support compared to the consenting group. Based on these findings, we argue that ignoring consent can have serious ethical ramifications for policy-making with nudges.

## Introduction

Behavioural nudges are light-touch signals that encourage citizens to make welfare-improving decisions, as judged by themselves and society^1^. Evidence suggests that nudging is cost-effective^2^. But at what cost? A common criticism is that nudges “work best in the dark”^3^, that is without people knowing that they are being nudged. This long-standing ethical critique to nudging rests on the covertness of some behavioural nudges. For example, defaults can bypass reasoning, encouraging individuals to choose an option quickly rather than carefully weighing up the arguments and evidence for and against doing the action^3,4^. After all, this is where nudges outshine traditional public policies as they do not require much effort on the part of individuals. This is problematic because people who are unaware of being nudged might be unduly influenced to make decisions contrary to what would have been their choice if they had been consulted^5–7^. When the stakes are high, it can be wrong, for example, to have elderly patients select a default of non-resuscitation if this discourages them from carefully considering their options. Even low-stakes decisions—such as deciding whether to donate a small sum to charity—are perhaps best made after careful reasoning rather than just from instinct, because careful reasoning is important for human agency. Building such agency is important for behavioural science^8^

One potential way to increase agency is to make nudges transparent: if individuals know they are being nudged, either before or during the nudge, they cannot be manipulated or discouraged from reasoning^9–11^. Experimental evidence suggests transparent nudges can also be equally effective in encouraging welfare-improving decisions^12–15^. However, these methods do not fully address the criticism that nudges reduce human agency^8,16^. For example, when an individual is told transparently that they are given a default to encourage donations, they may feel some pressure to donate an amount which they cannot quite afford or which is not warranted. They, therefore, might not be engaging in careful reasoning, perhaps even subject to manipulation despite being aware that they are being nudged^17^. Moreover, even if transparent nudges avoid manipulation or reason-bypassing, and have been found effective in some contexts, they may be ineffective in other contexts^18^. If and when they are ineffective, there is a trade-off to be made between instituting a non-transparent nudge to reach a given end—such as a fundraising goal—and instituting a transparent nudge to protect individuals from potential manipulation and the bypassing of reasons. In such cases, a question remains as to whether the non-transparent nudge is all-things-considered morally justified.

Another response to the criticism that nudges undermine agency is to evaluate whether most individuals endorse being nudged after the fact^19–22^, with evidence supporting that (most) people like nudges after all^23^. Nonetheless, nudges that are endorsed later (whether transparent or not) can still undermine autonomy by placing pressure on individuals which they cannot avoid. Moreover, many may not endorse having been nudged after the fact, even if most do.

In this paper, rather than relying on building transparency into nudges or seeking endorsement after the nudge, we propose a third alternative: making nudges consensual. We test these consensual nudges against non-consensual ones using our experimental design to answer the following research question: does violating consent to nudging reduce the effectiveness of the nudge and/or reduce human welfare?

Our findings contribute to a growing literature on the ethics of nudging. Our study is the first to test explicitly the effect of violating individual consent to nudging on behavioural outcomes and individual well-being. Moreover, the study has important policy implications for nudging people, and suggests that consensual nudging can become a potential remedy to the criticism of psychological manipulation. We discuss the experimental design in more detail in Section “Methods”. We summarise our findings in Section “Results” and discuss their implications in Section “Discussion”.

## Methods

### Survey design

We created an online survey experiment using Qualtrics and administered it to a sample of 1,518 United Kingdom citizens. The survey was preregistered on OSF (see here). The survey comprised four main sections. Initially, individuals were screened based on four pre-registered conditions: consent to the overall study, UK citizenship, age, and donation status in the last 12 months. Subsequently, participants underwent an attention check following Kalla and Broockman (Specifically, participants were asked: “People are very busy these days and many do not have time to follow what goes on in the government. We are testing whether people read questions. To show that you’ve read this much, answer both ‘Extremely interested’ and ‘Very interested’." Participants could then choose multiple options from a 7-point list ranging from extremely disinterested to extremely interested)^24^. Those meeting the inclusion criteria and passing the attention check (A total of 175 participants failed the attention check and were removed) proceeded to complete the survey.

In the second segment, individuals were asked to share their socio-economic beliefs and attitudes. Specifically, we inquired about their political ideology, generalised trust, preferred charities, beliefs regarding objectives and goals, altruism, positive and negative reciprocity, and perceived agency and autonomy. These pre-experimental covariates were pre-registered for use in a LASSO specification of our regression models. They were also used for matching individuals across consenting and non-consenting groups. For additional details, refer to the pre-analysis plan link. At the end of this segment, individuals were also asked for their consent to be nudged in making a welfare-improving choice. However, prior to this, we checked for attention of participants using a second attention screener (specifically, participants were asked: “Most modern theories of decision making recognize that decisions do not take place in a vacuum. Individual preferences and knowledge, along with situational variables can greatly impact the decision process. To demonstrate that you’ve read this much, just go ahead and select both red and green among the alternatives below, no matter what your favourite colour is. Yes, ignore the question below and select both of those options. What is your favourite colour?” Participants could choose multiple options from a list of 6 options: white, black, red, pink, green, blue. A total of 14 participants failed this attention check) following Berinsky and colleagues^25^. Individuals had the option of either stating that they did consent or not. When asking for consent, we included a brief explanation of what a nudge is, using default nudges as examples, including the UK policy if signing patients up as organ donors as a default, where patients can simply opt-out, and policies of charities signing individuals up to newsletters from which they can opt-out.

After seeking consent to nudging, in the third section individuals were randomly assigned to one of two experimental conditions using an A/B parallel between-subjects design. This default setup was varied randomly to avoid anchoring effects caused by a specific type of default presentation^26^. In both variants of the default, individuals were given the option of donating additional survey winnings to a charity of their choice with one sum ($\pounds$2) clearly marked as the default. They differed in the number of alternative options available to them besides the default sum. For example, in the case of the “single-choice default”, participants could donate either $\pounds$2 or opt-out and choose any other donation amount between $\pounds$0 and $\pounds$10. Contrarily, in the “multiple-choice default”, participants were shown multiple amounts ($\pounds$1, $\pounds$2, $\pounds$5, and $\pounds$10), with $\pounds$2 marked as the default, besides having the option to opt-out of the default and choosing any other amount as before. Each individual could choose a preferred charity from a list of over 200 organisations, picked according to popularity in the UK^27^ or effectiveness according to the “Give Well” rankings^28^. After selecting a charity, individuals were asked to indicate their preferred donation amount.

Finally, in the fourth section, following the donation choice, participants were provided with a disclosure about the nudge – those who had not consented were informed that they were nudged despite not having consented, while the remaining who had consented were informed that they were nudged as per their consent. All individuals were then asked if they would like to revise their donation amount following this disclosure and choose a revised amount (between $\pounds$0-$\pounds$10). Further, individuals were also asked to report their levels of happiness, resentment against the nudge, approval of the nudge, and regret for their donation using a Likert scale ranging between 0-10.

Because the study involved asking people if they consented to being nudged, and nudging them even if they did not consent, this involved a type of participant deception under experimental manipulation. This was done in line with the ethical guidelines of the research ethics committee of King′s College London. All participants were fully debriefed at the end of the study and were given an opportunity to finalise their donations after they were debriefed. Finally, participants reported standard demographics. At the end, they were provided with an opportunity to offer feedback in the form of open-ended text. The complete survey is available in the Supplementary Index.

### Power analysis and hypothesis

We conducted an ex-ante sampling analysis, pre-registering our intent to recruit a minimum of 1,500 participants from Prolific. This analysis was based on a two independent-groups, means-difference comparison (t-test). With a power of 80%, we set an ex-ante Bonferroni corrected type-1 error probability ($\alpha$) of 0.0125 (0.05/4 hypotheses) to detect a minimum effect size of d=0.173. Additionally, we assumed equal allocation across both treatment conditions, as recruitment costs were equivalent for participants in both groups. In summary, our study was adequately powered to identify minimum detectable effect sizes (MDES) equivalent to a small Cohen’s d (d$\ge$0.20).

We pre-registered the following confirmatory hypothesis:

**H1** Those who do not consent to nudging will produce greater differences in charitable donations [due to variations in process freedom] compared to those who consent. This is a one-sided test.

**H2** Those who do not consent will report greater resentment to the nudge compared to those who consent. This is a one-sided test.

**H3** Those who do not consent will report lower approval of the nudge compared to those who consent. This is a one-sided test.

In addition, we also tested the following two exploratory hypotheses with two additional outcomes.

**H4** Those who do not consent will report greater regret for donation under the nudge compared to those who consent. This is a one-sided test.

**H5** Those who do not consent will report lower happiness levels compared to those who consent. This is a one-sided test.

### Statistical analysis

Following our preregistration plan, we used standard linear regression models with heteroscedasticity—robust standard errors to estimate regression coefficients. We used the Least Absolute Shrinkage and Selection Operator (LASSO) to select covariates for prognostic analysis (using covariates reported in the pre-analysis plan). Further, we also matched individuals in the non-consenting group with individuals in the consenting group using the *psmatch2* package in STATA 17.1. We corrected for all confirmatory (and exploratory) tests reported above using the^29^ joint hypothesis testing (reporting the randomised t- p-values).

### Ethics approval

This research study was approved by the Research Ethics Committee of King’s College London (Ref—HR-22/23-29151.).

## Results

### Consent is irrelevant to the effectiveness of the default nudge

In our sample, 437 individuals (29%) did not consent to being nudged, whereas the remaining 1081 individuals (71%) consented. A majority of the participants in our sample, therefore, consented to being nudged. The probability to consent to being nudged (versus not consenting) was meaningfully higher for individuals in the sample with higher levels of trust (Odds Ratio (OR) = 1.35; p = 0.019), education (OR = 1.07, p = 0.096) and beliefs of ownership for decisions taken by charity (OR = 1.05, p = 0.071); and lower for participants who had right-leaning political beliefs (OR = 0.88, P< 0.0001). A full list of these odds ratios from the binary logistic regression is reported in Table A7 in the Supplementary Index).

We find that violating consent to nudging does not produce any meaningful difference in charitable donation amounts between consenting and non-consenting groups of individuals in the study. Individuals who consent to the nudge donate $\pounds$4.5 ($\sigma =3.2$), on average, to a preferred charity. We find 68% of consenting individuals stick to the default sum of donation and do not opt-out. Further, individuals who do not consent to being nudged ex-ante donate $\pounds$4.1 ($\sigma =3.0$), on average, to a charity of their choice. In this group, 66% stick to the default choice. The difference in average donation amounts between the consenting and the non-consenting group of individuals is small ($\mu =0.41, \sigma =0.25$) and not meaningful ($p=1.00$). Similarly, these groups do not differ meaningfully in their opt-out rates ($p=0.3834$).

**Figure 1:**
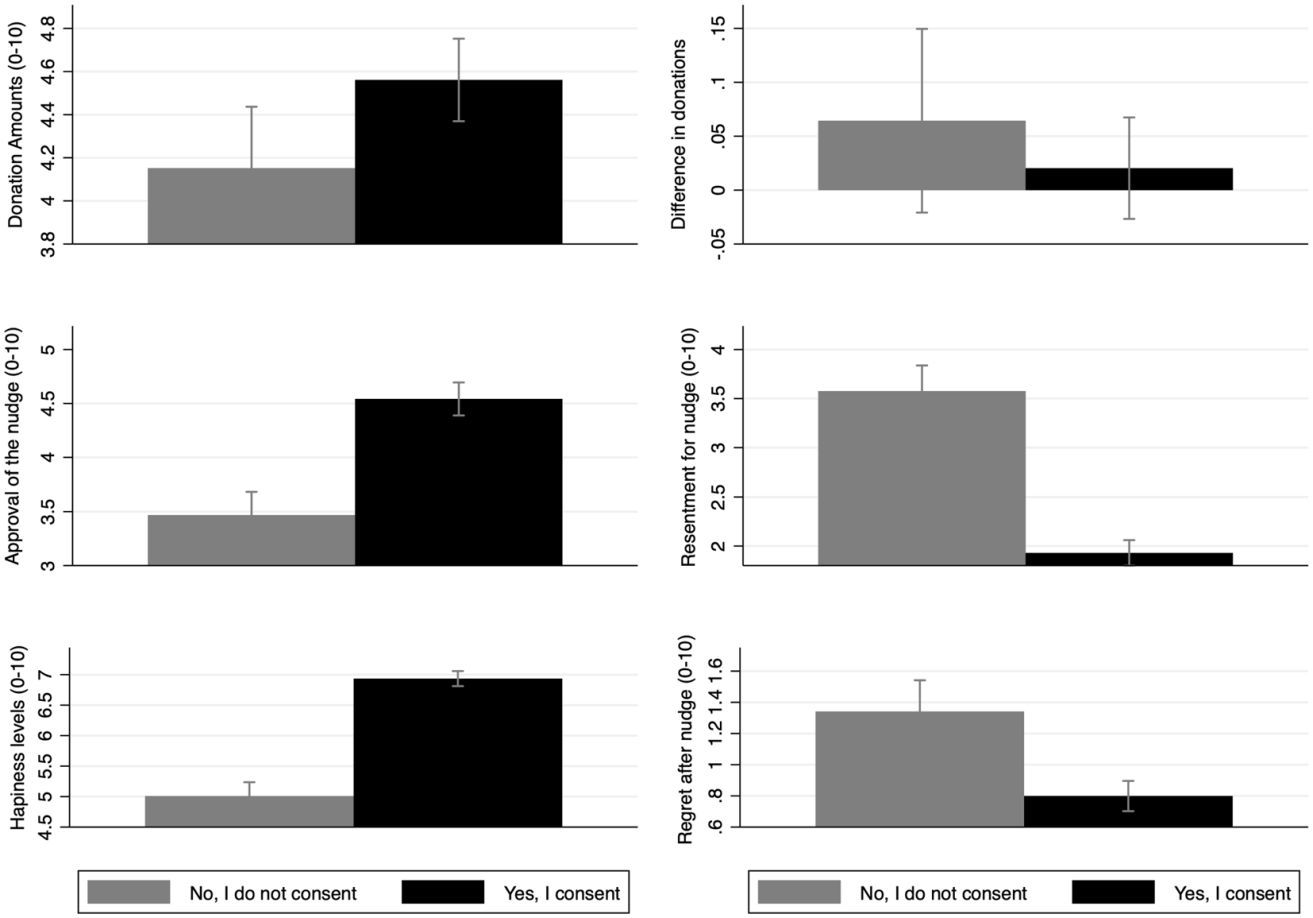
Each panel plots a (means) bar graph with 95% CIs for those who do not consent (gray bar) versus those who consent (black bar). Top-Left to Bottom Right: Panel 1: Charitable donations, pre-disclosure; Panel 2: Differences in charitable donations, pre and post disclosure; Panel 3: Approval of the nudge, post-disclosure; Panel 4: Resentment for nudge, post-disclosure; Panel 5: Happiness, post-disclosure; Panel 6: Regret, post-disclosure.

We also do not find any meaningful revision in donation amounts following the disclosure. Recall that individuals were debriefed after their initial donation choices about whether or not their consent was respected. On being told this, 7% of the non-consenting individuals indicated a willingness to revise their donation amount compared to 6% in the group of consenting individuals. The average donation amount was unchanged at $\pounds$4.5 ($\sigma =3.2$) and $\pounds$4.1 ($\sigma =3.1$) in the consenting and non-consenting group of individuals, respectively. There is no meaningful difference in these donation revisions following the disclosure between the consenting and non-consenting groups of individuals. Figure 1 (panels 1–2) plots these (raw) average donation amounts by consent types of individuals, before and after disclosure.

### Violating consent lowers welfare

Violating consent to nudging has negative connotations on certain aspects of public welfare. Recall that following a disclosure about the nudge, participants were asked to report measures of well-being (using a Likert scale ranging between 0-10), namely resentment against nudge, support for nudge, regret for their donation and happiness. We find that resentment against the nudge increases meaningfully by 11 percentage points ($\mu =1.3, p<0.00001$), on average, for individuals who did not consent to the nudge compared to consenting individuals. Similarly, public support for the nudge decreased by 8 percentage points ($\mu =0.85, p<0.00001$), on average, for non-consenting individuals versus those who consented. In addition, we also find that regret for the donation was meaningfully higher by 2 percentage points with non-consenting individuals ($\mu =0.26, p<0.1$) (however, this result does not hold when we control for covariates selectively using the LASSO, for details see Table 1). Finally, happiness levels of non-consenting individuals decreased meaningfully by 15 percentage points ($\mu =1.55, p<0.00001$) versus consenting individuals. Figure 1 plots the raw means of these well-being measures in panels 3–6.

**Table 1:** The effect of violating consent on behavioural outcomes.

	(1)	(2)	(3)	(4)	(5)	(6)
Panel A						
Consent to nudge	0.407	-0.007	-1.273***	0.854***	-0.215	1.554***
	(0.248)	(0.058)	(0.212)	(0.182)	(0.146)	(0.193)
	[0.1139]	[0.9105]	[0.00064]	[0.000006]	[0.1033]	[0.00037]
Controls (LASSO)	$\checkmark$	$\checkmark$	$\checkmark$	$\checkmark$	$\checkmark$	$\checkmark$
N	1458	1458	1483	1478	1480	1484
$R^2$	0.132	0.043	0.140	0.096	0.182	0.074
Panel B						
ATT	0.516	0.031	-1.555**	1.004**	-0.649**	1.854**
	(2.252)	(0.071)	(0.191)	(0.196)	(0.138)	(0.169)
N (On Support)	1455	1455	1450	1447	1452	1448

Columns 1–6 correspond to different outcome variables, namely donations, difference in donation post-disclosure, resentment, support, regret, and happiness. Panel A reports OLS estimates with heteroscedastic–robust standard errors in parentheses and Young’s^29^ randomised t- p-values in box-brackets; ***$p<0.01$, **$p<0.05$, *$p<0.1$ (raw p-values). Controls in regression were chosen using a LASSO linear regression specification. All columns control for the type of default and its interaction with consent. In addition, column 1 controls for political ideology, trust, beliefs about charity, negative and positive reciprocity, altruism, beliefs about ownership and decision-control when charities make decisions on their behalf, age, sex and education. Column 2 controls for political ideology, beliefs about charity, altruism and age. Column 3 controls for positive reciprocity, altruism and age. Column 4 controls for political ideology, trust, belief about charity (best interests), beliefs about ownership when charities make decisions on their behalf and age. Column 5 controls for political ideology, beliefs in charity (long-term interests), positive reciprocity, altruism, age, and education. Column 6 controls for trust, altruism, beliefs in charity (best interests), and beliefs about ownership and decision-control when charities make decisions on their behalf. Panel B reports Average Treatment Effects on Treated (1:1 matching) with standard errors in parentheses using psmatch2 package in STATA 17.1. The balancing property is satisfied for all six columns.

Randomising selection into consent is not plausible. In our study, individuals self-selected into consenting to the nudge or not. However, this could potentially lead to self-selection bias. To overcome this, we use two robustness specifications, first controlling for covariates selectively using the LASSO and correcting for multiple hypotheses using Young’s method^29^ and second matching individuals (on a 1:1 basis) who do not consent to those who consent based on pre-selection covariates. Panel A of table 1 reports the coefficients from linear regression models with LASSO in columns 1-6 whereas Panel B reports the Average Treatment Effect on Treated (ATT) following a 1:1 matching analysis. The findings across both panels are robust to our comparison of raw means reported above, with the exception of column 5 (regret) which is statistically significant only in the matching specification. In addition, the magnitude of the effect of violating consent on the behavioural outcomes increases following the matching analysis, suggesting some downward bias in estimation reported in Panel A. Figure A1 & A2 plots distribution of the propensity scores across consenting and non-consenting groups, before and after matching, for all outcomes (see Supplementary Index). Table A1–A6 reports the statistical balance of means between consenting and non-consenting individuals, across different observed covariates, after matching. More details on these statistical methods are available in the Methods.

## Discussion

In this paper, we find that nudges are effective even when individuals  consent to their execution. There is no meaningful difference in average contributions to charities between consenting and non-consenting groups. Moreover, telling individuals whether they have been nudged does not meaningfully change their indicative initial donation amounts, amongst both consenting and non-consenting individuals. However, non-consenting individuals report meaningfully higher levels of resentment against nudges and regret for their donation versus consenting individuals. In addition, they also report lower levels of happiness and public support for the nudge. This suggests that consensual nudges are not only compatible with being effective but also avoid certain moral hazards.

In asking individuals if they consented to being nudged, we introduced a method of building autonomy that is neither reducible to transparency—where individuals are told they are being nudged but never consent—but also compatible with non-transparency: consent requires merely that individuals are aware of what a nudge entails in general, and are only nudged after they agree to being nudged. Indeed, even if the nudges are manipulative and reason-bypassing (some of the ways that nudges can reduce autonomy), if individuals give consent then they are waiving their right to not be subject to such phenomena. Of course, some nudges, such as the educative ones, may not be manipulative or reason-bypassing at all—that is still under debate^30^—but if or when they are, then consent can mitigate or dissolve these worries. This is because, more generally, consent has the power to render certain acts permissible^31–35^. Just as taking somebody’s money and giving it to charity can be rendered permissible with the person’s consent, nudging someone in a manipulative and reason-bypassing way can be rendered permissible with a person’s consent. And just as a person can give consent without complete transparency about what specifically will occur^33^, as when an individual consents to hypnosis without being told exactly how this will be executed and what they will feel, an individual can consent to being nudged in general without being told exactly what the nudge will entail. If such consensual nudges are also effective at encouraging certain actions, then the tension between protecting autonomy and reaching certain goals is mitigated. The idea that nudges could, in theory, be explicitly consented to, and that some of the worries about manipulative nudges are avoided if consent is given, has appeared scantily in the literature (see^5^). We build on this and test explicitly if consensual nudges are effective as well.

Our experimental findings indicate that consensual nudges are effective at encouraging the selection of the default, and so if consent also increases autonomy, the trade-off between effectiveness and autonomy is somewhat mitigated. This is a welcome finding for those who both value bringing about certain welfare-improving outcomes and those that seek to increase individuals’ control over their choices when being nudged. The above findings also suggest that non-consenting individuals can feel resentment at being nudged not because the nudge effects their final decisions. Individuals in the study felt resentment despite not changing their final donation amount, perhaps because they did not wish to be placed in a position where others attempted to manipulate them or bypass their reasoning, even if such manipulation or reason-bypassing did not impact their decision-making. Alternatively, perhaps the nudge did impact their decision-making. Once the decision was made they struggled to undo the nudge’s effects; they felt potentially uncomfortable taking back a donation amount they had already been nudged into giving, and felt resentment at having been nudged without their consent. We cannot test this with our experimental design, but regardless: greater resentment was felt amongst those who did not consent, and this resentment may be tracking some type of moral wrong. If individuals have an interest and right in controlling whether they are nudged, this interest and right can be set back even if the nudge has no effect, or has an effect that one struggles to undo.

Our findings generate insights for organisations such as charities who are seeking to increase their received donations or in medical settings where patients could be made better off with certain defaults. In particular, our experimental insights provide good reason for organisations to ask individuals if they consent to being nudged before given a default. They can do so by simply asking individuals if they would like be nudged, especially after providing them information about what a nudge is, with a special focus on what a default is and why it is provided. If individuals consent to being nudged via a default before the default is provided, and are not nudged if they do not consent, then their autonomy can be better protected while maintaining some of the nudge’s effectiveness. Our findings are also potentially generalisable to cases of citizen or consumer manipulation, where there might be negative consequences of violating consent, such as through reduced trust in actors who are seen to manipulate. While we did not measure trust ex-post as an experimental outcome, we find that higher trust is negatively correlated with resentment levels (-0.49, p<0.001) of participants. Future research should assess the effect of consent and manipulation on social preferences.

Though we have demonstrated that the tension between autonomy and effectiveness can be reduced with consent, the tension is not reduced entirely. If individuals who do not consent to being nudged are not nudged into a default option, and less likely to select this option as a result, then fewer will select the option if nudges are limited to those who consent. Even if those who do consent will be more likely to select the default compared to alternatives, such that the nudge still has some effectiveness, simply nudging everyone regardless of consent may very well encourage even more to select the default. For example, charities which simply give a default of $\pounds$2 to everyone, regardless of who consents, may succeed in encouraging more to donate this amount than a charity that limits defaults to those who do consent. We cannot rule this possibility out from the findings we present, as we did not include a group that was not nudged after failing to consent. However, even if the tension between autonomy and reaching certain goals is not necessarily avoided, we have demonstrated that it is mitigated: consensual nudges are indeed effective, and so preserving autonomy via consent needn’t come at the complete expense of reaching certain goals via nudges. If so, then those concerned that nudges are manipulative and reason-bypassing can ask for consent to avoid these wrongs while still encouraging certain choices.

It is worth noting, however, that though asking for consent prior to a nudge can help avoid wronging people with manipulation and the bypassing of reason, it doesn’t necessarily reduce these phenomena themselves. Our claim is not that consent takes away manipulation or reasoning-bypassing, only that it makes these phenomenon less wrong. If so, individuals might withdraw their consent once they experience these phenomenon. For example, individuals might consent to being nudged, knowing what this entails, but upon seeing the default feel unwanted pressure to donate this amount, regretting their decision to consent to the nudge. They might therefore wish to withdraw their consent during this nudge, such as by asking that the default is removed or closing a browser if they are nudged online. In our survey, participants could withdraw consent by closing the browser at any time, and they were informed of this beforehand, but participants likely completed the survey (and indeed all did) to obtain the payment at the end. In real-world situations, incentives like a survey reward might not exist, and people might therefore withdraw consent more frequently during the nudge itself. This could affect outcomes like donation rates. On the other hand, if people feel comfortable withdrawing consent, they might feel in control and supportive of the nudge even if they aren’t given a reward like in our study. Further research—and ethical analysis as to remaining trade-offs between agency and reaching donation goals—are needed to explore these possibilities.

Our research also has some more general limitations. First, our findings are not completely causal—individuals self-selected into whether they consent or not to being nudged and there was no plausible way to randomise consent. Consequently, it is likely that consenting and non-consenting individuals might differ. We minimise observed bias by controlling for covariates using the LASSO and also by matching consenting individuals to non-consenting ones. However, individuals could differ in ways that were unobserved in our survey. A repeated survey (panel data) that accounts for individual fixed effects could overcome this limitation. Second, the response to consensual nudges could also vary by the source of the nudge. In the current study, individuals were informed that the nudge would be delivered by a group of researchers, but perhaps different responses would arise if the nudge was given by other types of groups, because trust in source can moderate findings. Third, we are unable to compare our findings to groups that are not asked for consent at all or groups who do not receive the disclosure following the nudge. Such comparisons can yield meaningful results into how consensual nudges work and future research should study these pathways. Despite these limitations, consensual nudging is a promising alternative to minimising the tension between effectiveness and autonomy and we call for more applications of consensual nudges.

### Supplementary Information

Supplementary Information.

## Data Availability

The data set generated during and analysed during the current study will be made available in OSF prior to publication.
