# Supplementary material for: Nudging against consent is effective but lowers welfare
Source: Sci Rep. 2024 Jun 27;14:14864. doi: 10.1038/s41598-024-65122-0 (PMC11211337; doi:10.1038/s41598-024-65122-0)
Supplement: Supplementary file 1 — Supplementary Information. [file 41598_2024_65122_MOESM1_ESM.pdf]

# Supplementary Index

## Additional Results

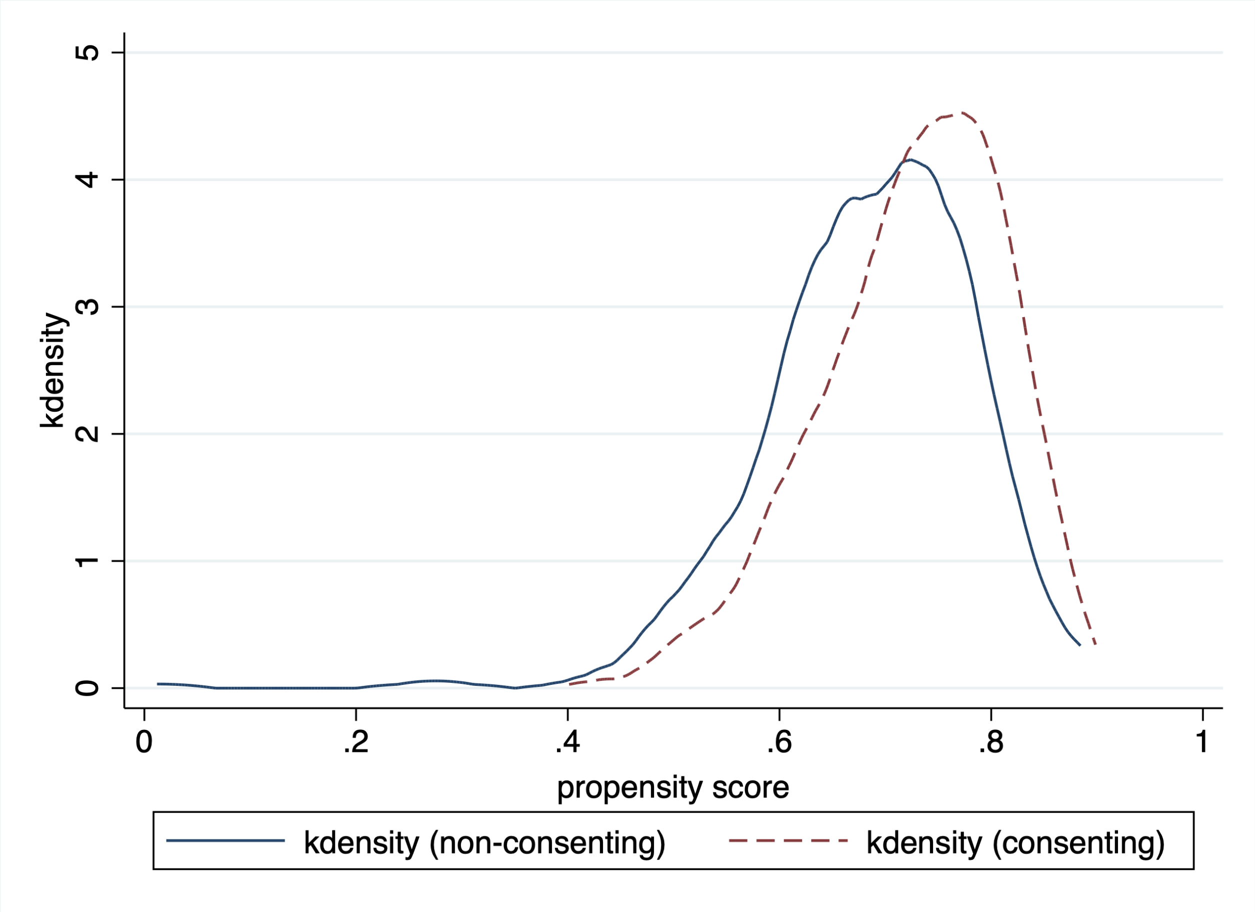


Figure A1: K-density plot of propensity scores across consenting and non-consenting groups of individuals prior to matching

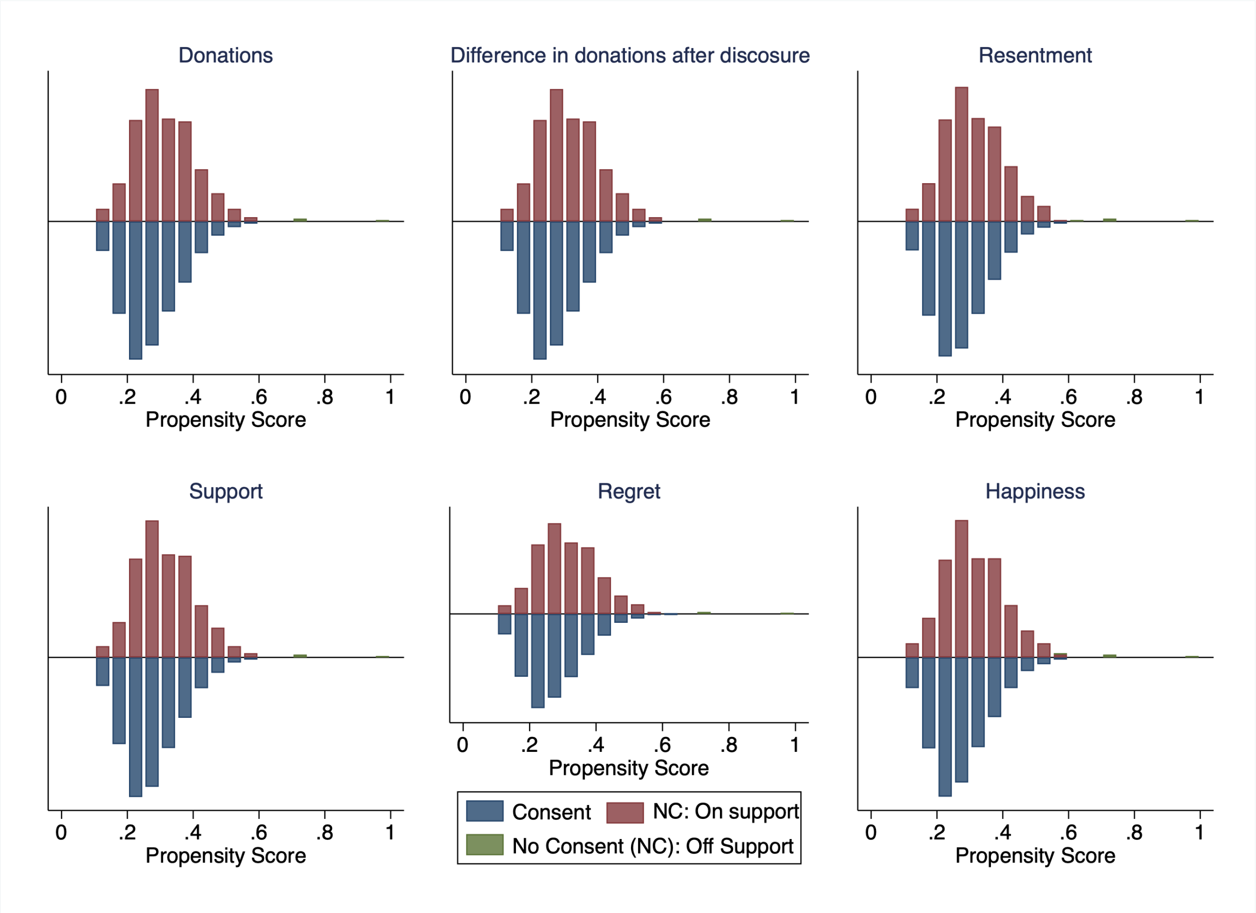


Figure A2: Propensity score graphs after matching for different behavioural outcomes

| Covariate | Non-Consenting | Consenting | % bias | t | p>t | V(C) |
|---|---|---|---|---|---|---|
| Political ideology | 43.976 | 4.347 | 2.5 | 0.36 | 0.721 | 1.09 |
| Trust | 0.45301 | .46747 | -2.9 | -0.42 | 0.677 | . |
| Charity | 52.795 | 54.554 | -5.9 | -0.84 | 0.404 | 1.02 |
| Charity (best interest) | 59.928 | 59.904 | 0.1 | 0.02 | 0.987 | 0.86 |
| Charity (ethical) | 56.289 | 52.988 | 10.6 | 0.86 | 0.393 | 0.08* |
| Charity (fair) | 57.349 | 55.036 | 7.3 | 0.59 | 0.553 | 0.08* |
| Charity (long-term interests) | 71.831 | 72.289 | -1.1 | -0.36 | 0.719 | 0.79* |
| Negative reciprocity | 47.036 | 44.169 | 11.8 | 1.70 | 0.090 | 0.98 |
| Altruism | 84.337 | 84.867 | -3.6 | -0.51 | 0.610 | 0.91 |
| Positive reciprocity | 86.024 | 85.639 | 2.6 | 0.36 | 0.720 | 0.73* |
| Ownership in charity decisions | 48.771 | 4.853 | 1.0 | 0.14 | 0.891 | 0.89 |
| Decision control in charity decisions | 60.072 | 60.675 | -1.5 | -0.47 | 0.642 | 0.93 |
| Age | 23.157 | 23.446 | -2.3 | -0.32 | 0.749 | 0.90 |
| Sex | .59036 | .55904 | 6.4 | 0.91 | 0.362 | . |
| Education | 14.875 | 14.822 | 3.4 | 0.46 | 0.644 | 0.86 |

Table A1: Balance of means test after matching, for Outcome: Donation

| Covariate | Non-Consenting | Consenting | % bias | t | p>t | V(C) |
|---|---|---|---|---|---|---|
| Political ideology | 43.976 | 4.347 | 2.5 | 0.36 | 0.721 | 1.09 |
| Trust | .45301 | .46747 | -2.9 | -0.42 | 0.677 | . |
| Charity | 52.795 | 54.554 | -5.9 | -0.84 | 0.404 | 1.02 |
| Charity (best interest) | 59.928 | 59.904 | 0.1 | 0.02 | 0.987 | 0.86 |
| Charity (ethical) | 56.289 | 52.988 | 10.6 | 0.86 | 0.393 | 0.08* |
| Charity (fair) | 57.349 | 55.036 | 7.3 | 0.59 | 0.553 | 0.08* |
| Charity (long-term interests) | 71.831 | 72.289 | -1.1 | -0.36 | 0.719 | 0.79* |
| Negative reciprocity | 47.036 | 44.169 | 11.8 | 1.70 | 0.090 | 0.98 |
| Altruism | 84.337 | 84.867 | -3.6 | -0.51 | 0.610 | 0.91 |
| Positive reciprocity | 86.024 | 85.639 | 2.6 | 0.36 | 0.720 | 0.73* |
| Ownership in charity decisions | 48.771 | 4.853 | 1.0 | 0.14 | 0.891 | 0.89 |
| Decision control in charity decisions | 60.072 | 60.675 | -1.5 | -0.47 | 0.642 | 0.93 |
| Age | 23.157 | 23.446 | -2.3 | -0.32 | 0.749 | 0.90 |
| Sex | .59036 | .55904 | 6.4 | 0.91 | 0.362 | . |
| Education | 14.875 | 14.822 | 3.4 | 0.46 | 0.644 | 0.86 |

Table A2: Balance of means test after matching, for Outcome: Difference in donations post disclosure

| Covariate | Non-Consenting | Consenting | % bias | t | p>t | V(C) |
|---|---|---|---|---|---|---|
| Political ideology | 43.786 | 43.447 | 1.6 | 0.24 | 0.812 | 1.04 |
| Trust | .45388 | .46359 | -1.9 | -0.28 | 0.780 | . |
| Charity | 52.816 | 53.519 | -2.4 | -0.33 | 0.740 | 1.00 |
| Charity (best interest) | 6 | 60.097 | -0.5 | -0.07 | 0.946 | 0.87 |
| Charity (ethical) | 56.359 | 53.592 | 8.9 | 0.93 | 0.351 | 0.14* |
| Charity (fair) | 5.733 | 55.995 | 4.2 | 0.45 | 0.655 | 0.15* |
| Charity (long-term interests) | 71.772 | 71.359 | 1.0 | 0.32 | 0.749 | 0.76* |
| Negative reciprocity | 47.136 | 45.437 | 7.0 | 0.99 | 0.324 | 0.91 |
| Altruism | 84.417 | 85.752 | -9.1 | -1.27 | 0.203 | 0.87 |
| Positive reciprocity | 86.165 | 86.432 | -1.8 | -0.25 | 0.799 | 0.77* |
| Ownership in charity decisions | 48.908 | 46.699 | 8.8 | 1.22 | 0.224 | 0.80* |
| Decision control in charity decisions | 60.049 | 58.932 | 2.7 | 0.82 | 0.410 | 0.80* |
| Age | 23.155 | 22.767 | 3.0 | 0.42 | 0.672 | 0.87 |
| Sex | .58981 | .61408 | -5.0 | -0.71 | 0.477 | . |
| Education | 14.876 | 14.883 | -0.5 | -0.06 | 0.949 | 0.87 |

Table A3: Balance of means test after matching, for Outcome: Resentment

| Covariate | Non-Consenting | Consenting | % bias | t | p>t | V(C) |
|---|---|---|---|---|---|---|
| Political ideology | 43.913 | 44.903 | -4.8 | -0.68 | 0.497 | 0.97 |
| Trust | .45411 | .47101 | -3.4 | -0.49 | 0.626 | . |
| Charity | 52.874 | 50.121 | 9.2 | 1.31 | 0.192 | 1.01 |
| Charity (best interest) | 59.903 | 61.812 | -9.6 | -1.36 | 0.173 | 0.96 |
| Charity (ethical) | 56.256 | 58.261 | -6.4 | -1.36 | 0.176 | 0.94 |
| Charity (fair) | 57.319 | 60.894 | -11.3 | -2.35 | 0.019 | 0.96 |
| Charity (long-term interests) | 71.836 | 72.536 | -1.7 | -0.58 | 0.564 | 0.95 |
| Negative reciprocity | 47.077 | 4.872 | -6.7 | -0.97 | 0.330 | 1.00 |
| Altruism | 8.43 | 85.072 | -5.2 | -0.72 | 0.474 | 0.79* |
| Positive reciprocity | 86.039 | 85.556 | 3.3 | 0.44 | 0.658 | 0.70* |
| Ownership in charity decisions | 48.816 | 4.942 | -2.4 | -0.35 | 0.729 | 0.92 |
| Decision control in charity decisions | 6 | 60.314 | -0.8 | -0.25 | 0.806 | 0.98 |
| Age | 23.188 | 23.382 | -1.5 | -0.22 | 0.828 | 0.97 |
| Sex | .58937 | .58454 | 1.0 | 0.14 | 0.888 | . |
| Education | 14.872 | 14.891 | -1.2 | -0.18 | 0.856 | 1.16 |

Table A4: Balance of means test after matching, for Outcome: Support

| Covariate | Non-Consenting | Consenting | % bias | t | p>t | V(C) |
|---|---|---|---|---|---|---|
| Political ideology | 43.961 | 4.314 | 4.0 | 0.56 | 0.576 | 0.95 |
| Trust | .45169 | .45894 | -1.5 | -0.21 | 0.834 | . |
| Charity | 52.802 | 4.971 | 10.4 | 1.46 | 0.145 | 1.00 |
| Charity (best interest) | 59.976 | 59.493 | 2.4 | 0.34 | 0.731 | 0.94 |
| Charity (ethical) | 56.329 | 51.473 | 15.6 | 1.26 | 0.209 | 0.08* |
| Charity (fair) | 57.391 | 51.763 | 17.8 | 1.45 | 0.147 | 0.08* |
| Charity (long-term interests) | 7.186 | 69.952 | 4.7 | 1.54 | 0.123 | 0.88 |
| Negative reciprocity | 46.981 | 45.531 | 6.0 | 0.88 | 0.381 | 1.08 |
| Altruism | 84.348 | 83.816 | 3.6 | 0.51 | 0.609 | 0.92 |
| Positive reciprocity | 86.039 | 85.894 | 1.0 | 0.14 | 0.887 | 0.91 |
| Ownership in charity decisions | 48.865 | 50.145 | -5.1 | -0.73 | 0.468 | 0.87 |
| Decision control in charity decisions | 60.072 | 60.773 | -1.7 | -0.53 | 0.595 | 0.88 |
| Age | 2.314 | 23.164 | -0.2 | -0.03 | 0.979 | 0.89 |
| Sex | .58937 | .58213 | 1.5 | 0.21 | 0.833 | . |
| Education | 14.872 | 14.826 | 2.9 | 0.40 | 0.689 | 0.86 |

Table A5: Balance of means test after matching, for Outcome: Regret

| Covariate | Non-Consenting | Consenting | % bias | t | p>t | V(C) |
|---|---|---|---|---|---|---|
| Political ideology | 43.835 | 43.835 | 0.0 | -0.00 | 1.000 | 1.11 |
| Trust | .45388 | .43932 | 2.9 | 0.42 | 0.675 | . |
| Charity | 52.767 | 53.422 | -2.2 | -0.31 | 0.756 | 1.03 |
| Charity (best interest) | 59.927 | 5.966 | 1.4 | 0.19 | 0.846 | 0.99 |
| Charity (ethical) | 56.383 | 57.913 | -4.9 | -1.05 | 0.292 | 1.00 |
| Charity (fair) | 57.282 | 58.568 | -4.1 | -0.85 | 0.393 | 1.01 |
| Charity (long-term interests) | 71.748 | 7 | 4.3 | 1.42 | 0.156 | 0.88 |
| Negative reciprocity | 47.112 | 44.102 | 12.4 | 1.79 | 0.074 | 1.01 |
| Altruism | 84.393 | 83.932 | 3.1 | 0.44 | 0.663 | 0.85 |
| Positive reciprocity | 85.995 | 85.947 | 0.3 | 0.05 | 0.961 | 0.98 |
| Ownership in charity decisions | 48.689 | 48.641 | 0.2 | 0.03 | 0.978 | 0.97 |
| Decision control in charity decisions | 60.073 | 59.053 | 2.5 | 0.80 | 0.427 | 0.99 |
| Age | 23.155 | 23.034 | 1.0 | 0.14 | 0.890 | 1.01 |
| Sex | .58981 | .59951 | -2.0 | -0.28 | 0.777 | . |
| Education | 14.881 | 15.066 | -11.7 | -1.82 | 0.068 | 1.49* |

Table A6: Balance of means test after matching for Outcome: Happiness

## Survey questionnaire

The survey questionnaire is provided below.

# Consent by Default Types

---

Start of Block: consent

JS

ethics
Thank you for taking the time to participate in this study. Please note that you must be 18+, must speak English fluently, must be a citizen of the United Kingdom, and must have donated at least 1 GBP to a charity in the last year to participate in this study, otherwise please don't partake. The study should take around 10 minutes to complete. In the study, you will be asked a series of questions about your preference for donations to a charity. You will be paid for your participation in the survey. Just make sure to read all the instructions carefully and try your best. NOTE: You can withdraw from the study at any stage without providing an explanation. Your privacy is very important, so we always use anonymised data. Results from this work may be written up for publication in a peer reviewed journal. However, individual data will never be published, and we will not hold personal identifiers. This project is in line with the ethical guidelines established by the Research Ethics Committee of King's College London. For more details about this research project, please see this information sheet.

If you have any questions you would like to ask before starting the survey, please feel free to contact Dr. Mollie Gerver, King's College London: mollie.gerver@kcl.ac.uk

Please note in this survey we would like to ask some questions that may be perceived as sensitive, such as gender, ethnicity, political orientation, and religion. Providing information in response to these questions is entirely voluntary and you may withdraw your consent at any time.

If you are happy to participate, please confirm your participation by consenting to all these conditions below. If you do not consent to these conditions below, you will be redirected to the end of the survey.

1. I confirm that I have read and understood the information sheet dated ${date://CurrentDate/FL} for the above project.
2. I consent voluntarily to be a participant in this project and understand that I can refuse to take part and can withdraw from the project at any time, without having to give a reason, up until completion of the survey.
3. I consent to the processing of my personal information for the purposes explained to me in the Information Sheet. I understand that such information will be handled under the terms of UK data protection law, including the UK General Data Protection Regulation (UK GDPR) and the Data Protection Act 2018.
4. I understand that my information may be subject to review by responsible individuals from the

College for monitoring and audit purposes.
5. I understand that confidentiality and anonymity will be maintained, and it will not be possible to identify me in any research outputs
6. I agree that the research team may use my data for future research.
7. I understand that I must not take part if I fall under the exclusion criteria as detailed in the information sheet and explained to me by the researcher.
8. I understand that I will have the opportunity (with a 1 in 50 chance) to win a Prolific bonus payment up to £10.

9. I understand that, during the course of the experiment, I will be asked if I would like to donate my experimental earnings to a charity of my choice.
10. I understand that I will be able to choose from a list of charities to donate to, and none of the charities are affiliated with the researchers and/or Prolific. If I decide to donate at the end of the experiment, these donations will be deducted from the Prolific bonus payment if this was successfully won in the experiment.
11. I understand that any money I might win from the Prolific bonus will be on top of the money I am paid from Prolific to take part in the experiment, and I will only be asked if I would like to donate part of my potential Prolific bonus.
12. I understand that my decision of whether to donate any or all of my Prolific bonus winnings will be collected as part of the data collection for the research.
13. I understand that I will be paid for my participation in the experiment whether I decide to donate to a charity of not.

| | Agree (1) |
|---|---|
| I have understood all the conditions above and consent to participate in the survey (1) | ○ |

*Skip To: End of Survey If Thank you for taking the time to participate in this study. Please note that you must be 18+,... != I have understood all the conditions above and consent to participate in the survey [ Agree ]*

Page Break

*

age What is your age? Please enter as a number (e.g., 25).

________________________________________________________________

*Skip To: End of Survey If Condition: What is your age? Please en... Is Less Than or Equal to 17. Skip To: End of Survey.*

Page Break

UKCitizen Are you a citizen of the United Kingdom?

- ◯ Yes (1)
- ◯ No (2)

*Skip To: End of Survey If Are you a citizen of the United Kingdom? = No*

Page Break

Madedonation Have you donated 1 GBP or more to a charity in the last 12 months?

- ○ Yes (1)
- ○ No (2)
- ○ I cannot remember (3)

*Skip To: End of Survey If Have you donated 1 GBP or more to a charity in the last 12 months? != Yes*

**End of Block: consent**

---

**Start of Block: Prolific ID**

Q310 What is your Prolific ID?
*Please note that this response should auto-fill with the correct ID*

________________________________________________________________

**End of Block: Prolific ID**

---

**Start of Block: captcha**

captcha Before you proceed to the survey, please complete the Captcha below.

**End of Block: captcha**

---

**Start of Block: Prescreener1**

sex What is your sex?

Note: Your answer is key to understanding trends in the population. This information will be used for balancing our sample between groups of people of different sexes.

- ○ Male (1)
- ○ Female (2)

Page Break

prescreener1 People are very busy these days and many do not have time to follow what goes on in the government. We are testing whether people read questions. To show that you've read this much, answer both “Extremely interested” and “Very interested”:

- Extremely disinterested (1)
- Very disinterested (2)
- Somewhat disinterested (3)
- Neither disinterested nor interested (4)
- Somewhat interested (5)
- Very interested (6)
- Extremely interested (7)

**End of Block: Prescreener1**

---

**Start of Block: Pretreat**

party_t Timing
First Click (1)
Last Click (2)
Page Submit (3)
Click Count (4)

partyID In politics, do you usually think of yourself as a(n):

- Conservative Party (1)
- Labour Party (2)
- Scottish National Party (3)
- Liberal Democrats (4)
- Democratic Unionist Party (5)
- Sinn Féin (6)
- Plaid Cymru (7)
- Social Democratic and Labour Party (8)
- Green Party (9)
- Alliance Party of Northern Ireland (10)
- Another party (specify): (11) ________________________________________________
- No party (12)
- Don't know (13)

*Display This Question:*

*If In politics, do you usually think of yourself as a(n): = Conservative Party*

*Or In politics, do you usually think of yourself as a(n): = Labour Party*

*Or In politics, do you usually think of yourself as a(n): = Scottish National Party*

*Or In politics, do you usually think of yourself as a(n): = Liberal Democrats*

*Or In politics, do you usually think of yourself as a(n): = Democratic Unionist Party*

*Or In politics, do you usually think of yourself as a(n): = Sinn Féin*

*Or In politics, do you usually think of yourself as a(n): = Plaid Cymru*

*Or In politics, do you usually think of yourself as a(n): = Social Democratic and Labour Party*

*Or In politics, do you usually think of yourself as a(n): = Green Party*

*Or In politics, do you usually think of yourself as a(n): = Alliance Party of Northern Ireland*

partyid_strength How strongly ${partyID/ChoiceGroup/SelectedChoices} do you feel?

- ○ Very strongly (1)
- ○ Fairly strongly (2)
- ○ Not very strongly (3)
- ○ Don't know (4)

---

*Display This Question:*

*If In politics, do you usually think of yourself as a(n): = Another party (specify):*

*And And In politics, do you usually think of yourself as a(n): Text Response Is Not Empty*

partyid_strength How strongly ${partyID/ChoiceTextEntryValue/2} do you feel?

- ○ Very strongly (1)
- ○ Fairly strongly (2)
- ○ Not very strongly (3)
- ○ Don't know (4)

---

Page Break

lr_scale In political matters, people talk of the '**left**' and the '**right**'. How would you place your views on this scale, generally speaking?

| | Left | | | | | | | | | Right |
|---|---|---|---|---|---|---|---|---|---|---|
| | 0 | 1 | 2 | 3 | 4 | 5 | 6 | 7 | 8 | 9 | 10 |
| 1 () | | | | | | | | | | | |

Page Break

trustGSS Generally speaking, would you say that most people can be trusted or that you need to be very careful in dealing with people?

- Most people can be trusted (1)
- Need to be very careful (2)

Page Break

pol_system On a scale of 0-10, where **0** means you have '**no confidence at all**' and a **10** means you have a '**great deal of confidence**', how much confidence do you have in the following institutions?

| | No confidence at all | | | | | | | | Great deal of confidence | | |
|---|---|---|---|---|---|---|---|---|---|---|---|
| | 0 | 1 | 2 | 3 | 4 | 5 | 6 | 7 | 8 | 9 | 10 |
| The government () | | | | | | | | | | | |
| Political parties () | | | | | | | | | | | |
| Media () | | | | | | | | | | | |
| Academics () | | | | | | | | | | | |

Page Break

preferred_charity What is your preferred cause for donating to a charity? Please choose all that apply.

- ▢ Animal welfare (1)
- ▢ Environmental conservation (2)
- ▢ Health services (awareness, disease prevention/cure research) (3)
- ▢ Refugee Support (5)
- ▢ Art and Culture conservation (6)
- ▢ Community development (7)
- ▢ Education charities (8)
- ▢ Reducing Homelessness (9)
- ▢ Reducing hunger (11)
- ▢ Other (Please specify) (10) ________________________________________________

Page Break

belief_charity On a scale of 0-10, where 0 means 'I do not agree at all' and 10 means 'I completely agree', how much do you agree with the following statements?

| | I do not agree at all | I completely agree |
|---|---|---|
| | 0 1 2 3 4 5 6 7 8 9 10 | |
| Charities deliver services in the best possible way () | | |
| Charities pursue long-term objectives () | | |
| People working in charities are ethical and not corrupt () | | |
| Charities are transparent () | | |
| Charities treat all beneficiaries fairly and do not discriminate. () | | |

Page Break

fairness_reciprocity On a scale of 0-10, where 0 means ‘Completely unwilling to do so’ and 10 means ‘Very willing to do so’, how willing are you to:

| | Completely unwilling to do so | | | | | | | | | | Very willing to do so |
|---|---|---|---|---|---|---|---|---|---|---|---|
| | 0 | 1 | 2 | 3 | 4 | 5 | 6 | 7 | 8 | 9 | 10 |
| Punish someone who treat others unfairly, even if there may be costs for you () | | | | | | | | | | | |
| Give to good causes without expecting anything in return () | | | | | | | | | | | |
| Return a favour () | | | | | | | | | | | |

Page Break

ownership Imagine one of the following parties made a decision that affected you. How likely are you to have an opportunity to voice your opinion? Please choose on a scale of 0-10, where 0 means 'Not at all likely' and 10 means 'Very likely'.

| | Not at all likely | | | | | | | Very likely | | | |
|---|---|---|---|---|---|---|---|---|---|---|---|
| | 0 | 1 | 2 | 3 | 4 | 5 | 6 | 7 | 8 | 9 | 10 |
| Your local government () | | | | | | | | | | | |
| Your national government () | | | | | | | | | | | |
| A commercial entity () | | | | | | | | | | | |
| Public institutions () | | | | | | | | | | | |
| Your preferred charity () | | | | | | | | | | | |

Page Break

decisioncontrol Imagine one of the following parties made a decision that affected you. How likely are you to agree with their decision? Please choose on a scale of 0-10, where 0 means 'Not at all likely' and 10 means 'Very likely'.

| | Not at all likely | | | | | | | | | Very likely | |
|---|---|---|---|---|---|---|---|---|---|---|---|
| | 0 | 1 | 2 | 3 | 4 | 5 | 6 | 7 | 8 | 9 | 10 |
| Your local government () | | | | | | | | | | | |
| Your national government () | | | | | | | | | | | |
| A commercial entity () | | | | | | | | | | | |
| Public institutions () | | | | | | | | | | | |
| Your preferred charity () | | | | | | | | | | | |

Page Break

End of Block: Pretreat

Start of Block: Prescreener2

Prescreener2 Most modern theories of decision making recognize that decisions do not take place in a vacuum. Individual preferences and knowledge, along with situational variables can greatly impact the decision process. To demonstrate that you’ve read this much, just go ahead and select both red and green among the alternatives below, no matter what your favourite colour is. Yes, ignore the question below and select both of those options.

What is your favourite colour?

- ☐ White (1)
- ☐ Black (2)
- ☐ Red (3)
- ☐ Pink (4)
- ☐ Green (5)
- ☐ Blue (6)

Page Break

preamble In this part, we will ask you to make a decision that relates to charities.

---

JS

consentnudge Do you consent to being nudged into making a decision we think is ethical? Don’t worry, we won’t sign you up to a newsletter or ask you to donate an organ.

**What is a nudge? Click here to find out**A nudge happens when people try to encourage you to act a certain way. They do this by presenting you with an offer in a certain way. However, they never require you to do anything or ban you from doing anything. They also never punish you in any way (such as by fining you), and never lie to you.

For example, (1) Companies will sometimes automatically sign you up for their newsletters. You can always opt-out right away, and you are never forced to sign up. The company is nudging you into signing up for the newsletter. (2) When you sign up to a new GP practice, they will automatically sign you up to donate your organs if you die. You can always opt-out, and nobody will force you to be on the list. (3) Supermarkets will sometimes automatically round up the total on your receipt, adding a few pence to donate to charity. They let you opt out if you want, and not donate these few pence to charity. These supermarkets are nudging you into donating to charity.

- ◯ Yes, I consent to being nudged (1)
- ◯ No, I do not consent to being nudged (2)

**End of Block: Prescreener2**

---

**Start of Block: Multiple Choice Opt-Out Default YCon**

charitychoice_YCon There are many charities in the UK that help people build safe, happy, and productive lives by contributing to the social good. You can choose to donate to one of these charities if you like. The money you donate will not come from your own money; instead, we will enrol you in a lottery where you have a 1 out of 50 chance of winning an additional £10 Prolific bonus payment.

**Please select your preferred choice of charity below**.

Simply start typing the name of your preferred charity and it will show up if it is in the list. If you would not like to donate to any of the charities below, please tell us another charity you would likely donate to by choosing the option 'Other'. In the next step, you can choose an amount you are willing to donate (including £0).
My preferred charity is (7)

▼ MACMILLAN CANCER SUPPORT (1) ... OTHER (123)

*Display This Question:*

*If There are many charities in the UK that help people build safe, happy, and productive lives by co... = OTHER*

othercharity_mcYcon You indicated that your preferred charity was not included in the list of charities we provided. Please type the name of your preferred charity in the UK.

________________________________________________________________

Page Break

donationamount_YCon How much are you willing to donate to the charity of your choice, if you end up winning the additional £10 Prolific bonus payment?

Please note, you will be asked to re-evaluate your donation towards the end of this survey. Please choose an amount now based on what you are likely to donate later. We will confirm your donation amount later once again, and any donation you make will be sent by us to your designated charity.

- ○ I do not want to donate (1)
- ○ 1 GBP (8)
- ○ 2 GBP (9)
- ○ 5 GBP (10)
- ○ 10 GBP (11)
- ○ I want to donate another amount (Please enter a numeric value between 0-10) (12) ________________________________________________

Page Break

mc_YCon Do you think you were nudged to donate a certain amount to a charity?

- ◯ Yes, I was nudged to donate a certain amount (1)
- ◯ No, I was not nudged to donate a certain amount (2)
- ◯ I do not know if I was nudged (3)

---

Page Break

preamble_YCon Earlier in the experiment we asked for your consent to being nudged. You did consent to be nudged, and so we nudged you. You were nudged because the suggested option was on £2, and you could opt-out. The nudge in this case worked as follows: you were presented with a choice to donate to a preferred charity, and we automatically set your default choice to £2. You could have opted out of this nudge by choosing any other amount. In general, if you are given a default option that you can opt-out of, then you are more likely to accept this option than if you not given any option as a default. If we had just presented you different amounts of money you could click on, but no amount was set as the default, then you would not have been nudged.

Page Break

revisedonation_YCon Considering our decision to nudge you, would you like to change your donation amount now?

○ Yes, I would like to change my donation amount (1)

○ No, I would not like to change my donation amount (2)

*Skip To: End of Block If Considering our decision to nudge you, would you like to change your donation amount now? = No, I would not like to change my donation amount*

Page Break

reviseddonation_Ycon Please choose you revised donation amount now. Please note, your previously chosen donation amount was ${donationamount_YCon/ChoiceGroup/SelectedChoicesTextEntry}

| | 0 1 2 3 4 5 6 7 8 9 10 |
|---|---|
| My revised donation is () | |

End of Block: Multiple Choice Opt-Out Default YCon

Start of Block: Single Choice Opt-Out Default YCon

charitychoice_YCon There are many charities in the UK that help people build safe, happy, and productive lives by contributing to the social good. You can choose to donate to one of these charities if you like. The money you donate will not come from your own money; instead, we will enrol you in a lottery where you have a 1 out of 50 chance of winning an additional £10 Prolific bonus payment.

**Please select your preferred choice of charity below**.

Simply start typing the name of your preferred charity and it will show up if it is in the list. If you would not like to donate to any of the charities below, please tell us another charity you would likely donate to by choosing the option 'Other'. In the next step, you can choose an amount you are willing to donate (including £0).
My preferred charity is (7)

▼ MACMILLAN CANCER SUPPORT (1) ... OTHER (123)

*Display This Question:*

*If There are many charities in the UK that help people build safe, happy, and productive lives by co... = OTHER*

othercharity_scYcon You indicated that your preferred charity was not included in the list of charities we provided. Please type the name of your preferred charity in the UK.

________________________________________________________________

Page Break

donationamount_YCon How much are you willing to donate to the charity of your choice, if you end up winning the additional £10 Prolific bonus payment?

**We have automatically chosen 2 GBP as your preferred donation amount. You can opt-out of this by choosing "I want to donate another amount".**

Please note, you will be asked to re-evaluate your donation towards the end of this survey. Please choose an amount now based on what you are likely to donate later. We will confirm your donation amount later once again, and any donation you make will be sent by us to your designated charity.

- ○ 2 GBP (13)
- ○ I want to donate another amount (Please enter a numeric value between 0-10) (14) ________________________________________________

Page Break

mc_YCon Do you think you were nudged to donate a certain amount to a charity?

- Yes, I was nudged to donate a certain amount (1)
- No, I was not nudged to donate a certain amount (2)
- I do not know if I was nudged (3)

Page Break

preamble_YCon Earlier in the experiment we asked for your consent to being nudged. You did consent to be nudged, and so we nudged you. You were nudged because the suggested option was on £2, and you could opt-out. The nudge in this case worked as follows: you were presented with a choice to donate to a preferred charity, and we automatically set your default choice to £2. You could have opted out of this nudge by choosing any other amount.  In general, if you are given a default option that you can opt-out of, then you are more likely to accept this option than if you not given any option as a default. If we had just presented you different amounts of money you could click on, but no amount was set as the default, then you would not have been nudged.

Page Break

revisedonation_YCon Considering our decision to nudge you, would you like to change your donation amount now?

- Yes, I would like to change my donation amount (1)
- No, I would not like to change my donation amount (2)

*Skip To: End of Block If Considering our decision to nudge you, would you like to change your donation amount now? = No, I would not like to change my donation amount*

Page Break

reviseddonation_YCon Please choose you revised donation amount now. Please note, your previously chosen donation amount was ${donationamount_YCon/ChoiceGroup/SelectedChoicesTextEntry} .

| | 0 | 1 | 2 | 3 | 4 | 5 | 6 | 7 | 8 | 9 | 10 |
|---|---|---|---|---|---|---|---|---|---|---|---|
| My revised donation is () | | | | | | | | | | | |

End of Block: Single Choice Opt-Out Default YCon

---

Start of Block: Post nudge_YCon

happy Are you happy or unhappy about having been nudged in this way? Please answer on a scale of 0-10, where 0 means 'not happy at all' and 10 means 'extremely happy'.

| | Not happy at all | | | | | | Extremely happy | | | | |
|---|---|---|---|---|---|---|---|---|---|---|---|
| | 0 | 1 | 2 | 3 | 4 | 5 | 6 | 7 | 8 | 9 | 10 |
| My choice is () | | | | | | | | | | | |

---

resentment Do you feel resentment about being nudged in this way? Please answer on a scale of 0-10, where 0 means 'no resentment at all' and 10 means 'very resentful'.

| | No resentment at all | | | | | | Very resentful | | | | |
|---|---|---|---|---|---|---|---|---|---|---|---|
| | 0 | 1 | 2 | 3 | 4 | 5 | 6 | 7 | 8 | 9 | 10 |
| My choice is () | | | | | | | | | | | |

---

support Will you approve or disapprove of your government's actions, if it nudges citizens to make better decisions in this way? Please answer on a scale of 0-10, where 0 means 'completely disapprove' and 10 means 'completely approve'.

| | Completely disapprove | | | | | | Completely approve | | | | |
|---|---|---|---|---|---|---|---|---|---|---|---|
| | 0 | 1 | 2 | 3 | 4 | 5 | 6 | 7 | 8 | 9 | 10 |

| | |
|---|---|
| My choice is () | |

preregret Do you regret your decision to donate to the charity? Please answer on a scale of 0-10, where 0 means ‘do not regret at all’ and 10 means ‘completely regretful’.

| | Do not regret at all | Completely regretful |
|---|---|---|
| | 0 1 2 3 4 5 6 7 8 9 10 | |
| My choice is () | | |

Page Break

debrief Thank you for informing us about your preferred donation amount and charity. If you are win the lottery, we will contribute your donation amount to your preferred charity. We also want to take this opportunity to explain to you the purpose of this study in more detail.

At the start of the survey, we explained what a nudge is, asked if you consent to being nudged, and you did consent. We then nudged you. This is because we asked if you wanted to donate money to a charity, with one sum of money the default, where you needed to put an amount of money other than the default to change the donation. This was a nudge, as people are more likely to donate a default amount compared to other amounts.

We also told you afterwards that we nudged you if you wanted us to nudge you and asked you if you wanted to change your hypothetical donation choice. The reason we asked for your consent first and then nudged you is that we wish to understand whether people feel less resentment about being nudged when they are nudged only after giving their consent. Whether you felt resentment or not, that is valuable information, as it can help researchers understand the impact of non-consensual nudges. The reason we informed you if we respected your decision to be nudged is that we wish to understand whether people feel less resentment about being nudged if they consent and their consent was respected by researchers.

We also wanted to find out whether people who are nudged into donating a specific amount are more likely to donate this amount, as compared to some other amount, regardless of whether they consented to being nudged. For this reason, we recorded whether or not you agreed to donate part of your donation (though we don't know who you are, as this is an anonymous survey). We wanted to find out whether those who - like you - did consent to being nudged and were nudged were more likely to donate the default amount compared to other amounts, or whether they were less likely to donate the default as compared to those who did not consent to being nudged.

If you are unhappy for your data to be included in the research as a result of the information within the debrief, you must exit the survey and not submit your response. It will not be possible to retrospectively withdraw your survey responses after submission due to the anonymous nature of participation.

**End of Block: Post nudge_YCon**

---

**Start of Block: Multiple Choice Opt-Out Default NCon**

choicecharity_NCon There are many charities in the UK that help people build safe, happy, and productive lives by contributing to the social good. You can choose to donate to one of these charities if you like. The money you donate will not come from your own money; instead, we will enrol you in a lottery where you have a 1 out of 50 chance of winning an additional £10 Prolific bonus payment.

**Please select your preferred choice of charity below**.

Simply start typing the name of your preferred charity and it will show up if it is in the list. If you would not like to donate to any of the charities below, please tell us another refugee charity you would likely donate to by choosing the option 'Other'. In the next step, you can choose an amount you are willing to donate (including £0).
My preferred charity is (7)

▼ MACMILLAN CANCER SUPPORT (1) ... OTHER (123)

---

*Display This Question:*

*If There are many charities in the UK that help people build safe, happy, and productive lives by co... = OTHER*

othercharity_mcNcon You indicated that your preferred charity was not included in the list of charities we provided. Please type the name of your preferred charity in the UK.

________________________________________________________________

---

Page Break

donationamount_NCon How much are you willing to donate to the charity of your choice, if you end up winning the additional £10 Prolific bonus payment?

Please note, you will be asked to re-evaluate your donation towards the end of this survey. Please choose an amount now based on what you are likely to donate later. We will confirm your donation amount later once again, and any donation you make will be sent by us to your designated charity.

- ○ I do not want to donate (1)
- ○ 1 GBP (8)
- ○ 2 GBP (9)
- ○ 5 GBP (10)
- ○ 10 GBP (11)
- ○ I want to donate another amount (Please enter a numeric value between 0-10) (12) ________________________________________________

Page Break

mc_NCon Do you think you were nudged to donate a certain amount to a charity?

- ○ Yes, I was nudged to donate a certain amount (1)
- ○ No, I was not nudged to donate a certain amount (2)
- ○ I do not know if I was nudged (3)

---

Page Break

preamble_NCon Earlier in the experiment we asked for your consent to being nudged. You did not consent to be nudged. We nudged you anyhow. You were nudged because the suggested option was on £2, and you could opt-out. The nudge in this case worked as follows: you were presented with a choice to donate to a preferred charity, and we automatically set your default choice to £2. You could have opted out of this nudge by choosing any other amount. In general, if you are given a default option that you can opt-out of, then you are more likely to accept this option than if you not given any option as a default. If we had just presented you different amounts of money you could click on, but no amount was set as the default, then you would not have been nudged.

Page Break

revisedonation_NCon Considering our decision to nudge you, would you like to change your donation amount now?

- ○ Yes, I would like to change my donation amount (1)
- ○ No, I would not like to change my donation amount (2)

---

Page Break

reviseddonation_NCon Please choose you revised donation amount now. Please note, your previously chosen donation amount was ${donationamount_NCon/ChoiceGroup/SelectedChoicesTextEntry}.

| | 0 1 2 3 4 5 6 7 8 9 10 |
|---|---|
| My revised donation is () | |

End of Block: Multiple Choice Opt-Out Default NCon

Start of Block: Single Choice Opt-Out Default NCon

charitychoice_NCon There are many charities in the UK that help people build safe, happy, and productive lives by contributing to the social good. You can choose to donate to one of these charities if you like. The money you donate will not come from your own money; instead, we will enrol you in a lottery where you have a 1 out of 50 chance of winning an additional £10 Prolific bonus payment.

**Please select your preferred choice of charity below**.

Simply start typing the name of your preferred charity and it will show up if it is in the list. If you would not like to donate to any of the charities below, please tell us another refugee charity you would likely donate to by choosing the option 'Other'. In the next step, you can choose an amount you are willing to donate (including £0).
My preferred charity is (7)

▼ MACMILLAN CANCER SUPPORT (1) ... OTHER (123)

*Display This Question:*

*If There are many charities in the UK that help people build safe, happy, and productive lives by co... = OTHER*

othercharity_scNcon You indicated that your preferred charity was not included in the list of charities we provided. Please type the name of your preferred charity in the UK.

________________________________________________________________

Page Break

donationamount_NCon How much are you willing to donate to the charity of your choice, if you end up winning the additional £10 Prolific bonus payment?

**We have automatically chosen 2 GBP as your preferred donation amount. You can opt-out of this by choosing "I want to donate another amount".**

Please note, you will be asked to re-evaluate your donation towards the end of this survey. Please choose an amount now based on what you are likely to donate later. We will confirm your donation amount later once again, and any donation you make will be sent by us to your designated charity.

○ 2 GBP (13)

○ I want to donate another amount (Please enter a numeric value between 0-10) (14)
________________________________________________

Page Break

mc_NCon Do you think you were nudged to donate a certain amount to a charity?

- ◯ Yes, I was nudged to donate a certain amount (1)
- ◯ No, I was not nudged to donate a certain amount (2)
- ◯ I do not know if I was nudged (3)

Page Break

preamble_NCon Earlier in the experiment we asked for your consent to being nudged. You did not consent to be nudged. We nudged you anyhow. You were nudged because the suggested option was on £2, and you could opt-out. The nudge in this case worked as follows: you were presented with a choice to donate to a preferred charity, and we automatically set your default choice to £2. You could have opted out of this nudge by choosing any other amount. In general, if you are given a default option that you can opt-out of, then you are more likely to accept this option than if you not given any option as a default. If we had just presented you different amounts of money you could click on, but no amount was set as the default, then you would not have been nudged.

Page Break

revisedonation_NCon Considering our decision to nudge you, would you like to change your donation amount now?

- Yes, I would like to change my donation amount (1)
- No, I would not like to change my donation amount (2)

*Skip To: End of Block If Considering our decision to nudge you, would you like to change your donation amount now? = No, I would not like to change my donation amount*

Page Break

reviseddonation_NCon Please choose you revised donation amount now. Please note, your previously chosen donation amount was ${donationamount_NCon/ChoiceGroup/SelectedChoicesTextEntry}.

| | 0 1 2 3 4 5 6 7 8 9 10 |
|---|---|
| My revised donation is () | |

End of Block: Single Choice Opt-Out Default NCon

Start of Block: Post nudge_NCon

happy Are you happy or unhappy about having been nudged in this way? Please answer on a scale of 0-10, where 0 means ‘not happy at all’ and 10 means ‘extremely happy’.

| | Not happy at all Extremely happy<br>0 1 2 3 4 5 6 7 8 9 10 |
|---|---|
| My choice is () | |

resentment Do you feel resentment about being nudged in this way? Please answer on a scale of 0-10, where 0 means ‘no resentment at all’ and 10 means ‘very resentful’.

| | No resentment at all Very resentful<br>0 1 2 3 4 5 6 7 8 9 10 |
|---|---|
| My choice is () | |

approve Will you approve or disapprove of your government's actions, if it nudges citizens to make better decisions in this way? Please answer on a scale of 0-10, where 0 means ‘completely disapprove’ and 10 means ‘completely approve’.

Completely disapprove Completely approve

0 1 2 3 4 5 6 7 8 9 10

| | |
|---|---|
| My choice is () | |

regret Do you regret your decision to donate to the charity? Please answer on a scale of 0-10, where 0 means ‘do not regret at all’ and 10 means ‘completely regretful’.

| | Do not regret at all | Completely regretful |
|---|---|---|
| | 0 1 2 3 4 5 6 7 8 9 10 | |
| My choice is () | | |

Page Break

debrief Thank you for informing us about your preferred donation amount and charity. If you are win the lottery, we will contribute your donation amount to your preferred charity. We also want to take this opportunity to explain to you the purpose of this study in more detail.

At the start of the survey, we explained what a nudge is, asked if you consent to being nudged, and you did not consent. We then nudged you anyhow: we asked if you wanted to donate money to a charity, with one sum of money the default, where you needed to put an amount of money other than the default to change the donation. This was a nudge, as people are more likely to donate a default amount compared to other amounts.

We also told you afterwards that we nudged you even if you did not want us to nudge you and asked you if you wanted to change your hypothetical donation choice. The reason we nudged you, even though you did not consent to being nudged, is that we wish to understand whether people feel resentment when they are nudged without their consent. Whether you felt resentment or not, that is valuable information, as it can help researchers understand the impact of non-consensual nudges. The reason we informed you if we did not respect your decision to be nudged is that we wish to understand whether people feel more resentment about being nudged if they did not consent and their consent was not respected by researchers.

We also wanted to find out whether people who are nudged into donating a specific amount are more likely to donate this amount, as compared to some other amount, regardless of whether they consented to being nudged. For this reason, we recorded whether or not you agreed to donate part of your donation (though we don't know who you are, as this is an anonymous survey). We wanted to find out whether those who did consent to being nudged and were nudged were more likely to donate the default amount compared to other amounts, or whether they were less likely to donate the default as compared to those who - like you - did not consent to being nudged.

If you are unhappy for your data to be included in the research as a result of the information within the debrief, you must exit the survey and not submit your response. It will not be possible to retrospectively withdraw your survey responses after submission due to the anonymous nature of participation.

**End of Block: Post nudge_NCon**

---

**Start of Block: Demographics**

country Do you currently live in the United Kingdom?

○ Yes (1)

○ No (2)

Page Break

*Display This Question:*

*If Do you currently live in the United Kingdom? = Yes*

subnat_region Which region do you currently live in?

- ◯ East Anglia (1)
- ◯ East Midlands (3)
- ◯ London (4)
- ◯ North East (5)
- ◯ North West (6)
- ◯ Northern Ireland (7)
- ◯ Scotland (8)
- ◯ South East (9)
- ◯ South West (10)
- ◯ Wales (11)
- ◯ West Midlands (12)
- ◯ Yorkshire & Humberside (13)
- ◯ Prefer not to answer (2)

---

Page Break

Page Break

education What is the highest level of education you have achieved?

○ Combined Junior and Infant School/ Infant School (1)

○ Junior School (11)

○ Comprehensive School (12)

○ Comprehensive School (GCSE)/ Secondary Modern (GCSE)/ Grammar School (GCSE)/ City Technology College (GCSE)/ Sixth Form (13)

○ College/ Institution of Higher education (14)

○ Open College - College of Technology - Institute/ Teacher Training College (15)

○ University/ Open University (16)

○ Prefer not to answer (2)

Page Break

ethnicity Which of the following best describes your ethnicity?

- ☐ White: British (1)
- ☐ White: Irish (2)
- ☐ White: Other (3)
- ☐ Mixed: White and Black Caribbean (4)
- ☐ Mixed: White and Black African (5)
- ☐ Mixed: White and Asian (6)
- ☐ Mixed: Other mixed background (7)
- ☐ Black or Black British: African (8)
- ☐ Black or Black British: Caribbean (9)
- ☐ Black or Black British: Any other Black background (10)
- ☐ Asian or Asian British: Indian (11)
- ☐ Asian or Asian British: Pakistani (12)
- ☐ Asian or Asian British: Bangladeshi (13)
- ☐ Asian or Asian British: Other Asian background (14)
- ☐ Chinese (15)
- ☐ Other ethnic group not represented by these options (please specify) (16) ________________________________________________
- ☐ Do not wish to say (17)

Page Break

employment What is your employment status? Please select as many as applicable.

- ☐ Working for pay full-time (1)
- ☐ Working for pay part-time (2)
- ☐ Self-employed (3)
- ☐ Retired (4)
- ☐ Unemployed / Looking for work (5)
- ☐ Student (6)
- ☐ Caring for family (7)
- ☐ Other (Please specify) (8) ________________________________________________

Page Break

income What was your total household income, before taxes, for the year 2022?

- ○ No income (1)
- ○ £1 - £4,400 (2)
- ○ £4,401 - £8,800 (4)
- ○ £8,801 - £17,600 (5)
- ○ £17,601 - £26,400 (6)
- ○ £26,401 - £35,200 (7)
- ○ £35,201 - £52,800 (8)
- ○ £52,801 - £64,500 (9)
- ○ £64,501 - £88,000 (10)
- ○ £88,001 - £117,300 (11)
- ○ More than £117,300 (12)
- ○ Don't know / prefer not to answer (3)

---

Page Break

urban_rural Which of the following best describes the place where you now live…

- A large city (1)
- A suburb near a large city (2)
- A small city (3)
- A town (4)
- A rural area (5)

Page Break

religiosity In your life, you would say religion is:

- ◯ Very important (1)
- ◯ Somewhat important (2)
- ◯ Not very important (3)
- ◯ Not at all important (4)

End of Block: Demographics

---

Start of Block: feedback

feedback Do you have any comments on the survey? Please let us know about any problems you had or aspects of the survey that were confusing.

________________________________________________________________

________________________________________________________________

________________________________________________________________

________________________________________________________________

________________________________________________________________

End of Block: feedback

---

| Consent to being nudged | Odds ratio | Std. err. | z | P>z | [95% conf. | interval] |
|---|---|---|---|---|---|---|
| Political Ideology (Left-right) | .8832823 | .0265252 | -4.13 | 0.000 | .8327943 | .9368312 |
| Trust | 135.011 | .1722679 | 2.35 | 0.019 | 105.138 | 1.733.719 |
| Charity | 101.742 | .0203881 | 0.86 | 0.389 | .9782343 | 1.058.175 |
| Charity (Best) | 1.009.421 | .0371833 | 0.25 | 0.799 | .9391118 | 1.084.994 |
| Charity (Ethical) | 1.015.934 | .0343099 | 0.47 | 0.640 | .9508651 | 1.085.456 |
| Charity (Fair) | .9931095 | .03373 | -0.20 | 0.839 | .9291522 | 1.061.469 |
| Charity (Long term) | 101.473 | .0195693 | 0.76 | 0.448 | .9770905 | 1.053.819 |
| Negative Reciprocity | 1.012.073 | .0256129 | 0.47 | 0.635 | .9630968 | 1.063.539 |
| Altruism | 1.034.415 | .04849 | 0.72 | 0.470 | .9436112 | 1.133.956 |
| Positivreciprocity | 1.024.526 | .0463127 | 0.54 | 0.592 | .9376597 | 111.944 |
| Ownership in decisions by charity | 1.047.441 | .0269338 | 1.80 | 0.071 | .9959595 | 1.101.583 |
| Control in decisions by Charity | 1.042.024 | .0327347 | 1.31 | 0.190 | .9798003 | 1.108.199 |
| Age | .9460882 | .0473519 | -1.11 | 0.268 | .8576869 | 1.043.601 |
| Seks | 1.146.955 | .1426892 | 1.10 | 0.270 | .8987745 | 1.463.665 |
| Education | 1.065.154 | .0404261 | 1.66 | 0.096 | .9887956 | 1.147.409 |
| Constant | .3733621 | .2737539 | -1.34 | 0.179 | .0887193 | 1.571.239 |

Table A7: Binary logistic regression predicting consent to being nudged by pre-treatment covariates
